# Vatalanib for metastatic gastrointestinal stromal tumour (GIST) resistant to imatinib: final results of a phase II study

**DOI:** 10.1038/bjc.2011.151

**Published:** 2011-05-03

**Authors:** H Joensuu, F De Braud, G Grignagni, T De Pas, G Spitalieri, P Coco, C Spreafico, S Boselli, F Toffalorio, P Bono, T Jalava, C Kappeler, M Aglietta, D Laurent, P G Casali

**Affiliations:** 1 Department of Oncology, University Central Hospital of Helsinki, Haartmaninkatu 4, Helsinki FIN-00029, Finland; 2 Department of Medicine, European Institute of Oncology, Milano 20141, Italy; 3 Medical Oncology Unit, Istitute for Cancer Research and Treatment, Candiolo, Torino 10060, Italy; 4 Department of Cancer Medicine, Istituto Nazionale Tumori, Milano 20133, Italy; 5 Bayer Schering Pharma AG, Berlin 13353, Germany

**Keywords:** gastrointestinal stromal tumour, vatalanib, imatinib, sunitinib, tyrosine kinase inhibitor, targeted therapy

## Abstract

**Background:**

Vatalanib (PTK787/ZK 222584) inhibits a few tyrosine kinases including KIT, platelet-derived growth factor receptors (PDGFRs) and vascular endothelial growth factor receptors (VEGFRs). We report efficacy and safety results of vatalanib in advanced gastrointestinal stromal tumour (GIST) resistant to imatinib or both imatinib and sunitinib.

**Patients and methods:**

Forty-five patients whose metastatic GIST had progressed on imatinib were enrolled. Nineteen (42.2%) patients had received also prior sunitinib. Vatalanib 1250 mg was administered orally daily.

**Results:**

Eighteen patients (40.0% 95% confidence interval (CI), 25.7–54.3%) had clinical benefit including 2 (4.4%) confirmed partial remissions (PR; duration, 9.6 and 39.4 months) and 16 (35.6%) stabilised diseases (SDs; median duration, 12.5 months; range, 6.0–35.6+ months). Twelve (46.2%) out of the 26 patients who had received prior imatinib only achieved either PR or SD compared with 6 (31.6%, all SDs) out of the 19 patients who had received prior imatinib and sunitinib (*P*=0.324). The median time to progression was 5.8 months (95% CI, 2.9–9.5 months) in the subset without prior sunitinib and 3.2 (95% CI, 2.1–6.0) months among those with prior imatinib and sunitinib (*P*=0.992). Vatalanib was generally well tolerated.

**Conclusion:**

Vatalanib is active despite its narrow kinome interaction spectrum in patients diagnosed with imatinib-resistant GIST or with imatinib and sunitinib-resistant GIST.

Imatinib mesylate is currently regarded as the standard first-line treatment of advanced gastrointestinal stromal tumour (GIST), since the responses achieved are usually durable and imatinib is generally relatively well tolerated (Demetri *et al*, 2002; Verweij *et al*, 2004). Unfortunately, most GISTs ultimately progress despite continued imatinib administration, the median time to disease progression being ∼2 years as calculated from the date of initiation of therapy (Blanke *et al*, 2008). Sunitinib malate has been approved as the second-line treatment option for patients who do not tolerate imatinib or whose disease progresses on imatinib. In this setting, the objective response rate to sunitinib was 7% and the median time to progression (TTP) 6.3 months in a randomised prospective clinical trial that compared single-agent sunitinib with placebo (Demetri *et al*, 2006). When sunitinib is administered at the registered dose, 50 mg daily for 4 weeks followed by a 2-week break, or at a continuous dose of 37.5 mg daily, it is frequently associated with adverse effects that include mucositis, fatigue, diarrhea, hand–foot skin reaction, hypothyroidism and cardiac toxicity (Demetri *et al*, 2006). There is a need for well-tolerated and effective treatments for GIST patients whose disease ceases to respond to imatinib.

Vatalanib (PTK787/ZK 222584; Bayer Schering Pharma AG, Berlin; Novartis, East Hanover, NJ, USA) is an oral tyrosine kinase inhibitor (TKI) that inhibits the two key kinases that are often mutated in GIST, KIT and the platelet-derived growth factor receptor-*α* (PDGFR*α*), and all three isoforms of the vascular endothelial growth factor receptors (VEGFR-1 [Flt-1], VEGFR-2 [KDR] and VEGFR-3 [Flt-4]) (Wood *et al*, 2000). Vatalanib may thus be effective in the treatment of GIST. Imatinib is an inhibitor of several kinases including KIT, PDGFR*α* and -*β*, bcr-Abl, Abl, Arg and the colony-stimulating factor-1 receptor (FMS), and similarly sunitinib inhibits many kinases, including KIT, PDGFR*α*, PDGFR*β*, CSF-1R, Fms-like tyrosine kinase-3 receptor (FLT-3), the receptor encoded by the *ret* proto-oncogene (RET), and VEGFR-1, VEGFR-2 and VEGFR-3. In a study conducted by Karaman *et al* (2008), the interaction of vatalanib with the human kinome was more limited compared with imatinib, sunitinib, sorafenib or dasatinib, which suggests that vatalanib might be well tolerated. Since vatalanib blood level reaches peak concentration (*T*_max_) in 1.0–2.5 h and it has a short a half-life of 4.5 h, it does not accumulate at the steady state with once-daily (QD) dosing.

We have earlier reported treatment results with vatalanib of 15 patients diagnosed with advanced GIST whose disease progressed on imatinib (Joensuu *et al*, 2008). Vatalanib administered at the dose of 1250 mg orally QD was found active; two (13%) out of the 15 patients achieved partial remission (PR) and eight (53%) had stable disease (SD) ⩾3 months, and the median TTP was 8.5 months. As predicted by its limited kinome interaction profile, vatalanib was well tolerated. Here, we report the treatment results of 45 patients whose GIST progressed on imatinib and often also on sunitinib. We observed clinical activity of vatalanib in these patient populations, suggesting that broad kinase inhibition spectrum inhibitors may not always be necessary for attaining clinical activity in imatinib-resistant GIST.

## Patients and methods

### Patients

Patients were eligible to this phase II, prospective, open-label, multicentre (four centres) study provided that they had histologically, cytologically or radiologically confirmed metastatic GIST with at least one measurable tumour lesion as determined by Response Evaluation Criteria in Solid Tumours (RECIST) v. 1.0 (Therasse *et al*, 2000). We required that GIST was resistant to imatinib (objective evidence of progression after ⩾4 weeks’ treatment) or that the patient did not tolerate imatinib. Further inclusion criteria were age ⩾18 years, a World Health Organization performance status of 0–2, blood neutrophil count (ANC) ⩾1.5 × 10^9^ l^–1^, platelet count ⩾100 × 10^9^ l^–1^, haemoglobin ⩾9.0 g dl^–1^, aspartate aminotransferase and alanine aminotransferase ⩽3.0 × upper limit of normal (ULN) (⩽5.0 × ULN if liver metastases were present), total urinary protein collected in 24 h ⩽500 mg, serum creatinine ⩽1.5 × ULN, 24 h creatinine clearance ⩾50 ml per minute and life expectancy over 3 months. Patients were excluded if they received chemotherapy <4 weeks before study entry, had received a cumulative dose of doxorubicin >560 mg m^–2^ or epirubicin >800 mg m^–2^, had acute or chronic liver disease or were taking warfarin sodium.

The study protocol allowed expansion of the trial if clinical efficacy was observed in more than three out of the first 15 patients entered in the study, which condition was met (Joensuu *et al*, 2008). The expanded protocol allowed inclusion of also patients who had progressed on both imatinib and sunitinib. Patients who had received another KDR inhibitor therapy than sunitinib were excluded. None of the patients had received adjuvant imatinib.

Written informed consent was obtained from each patient before study inclusion. The protocol was approved by an Independent Ethics Committee, and each investigator conducted the trial (ClinicalTrials.gov identifier NCT00117299) in accordance with the Declaration of Helsinki.

### Vatalanib administration

Vatalanib 1250 mg (five 250 mg tablets) was administered orally QD on a continuous basis until progressive disease (PD) or unacceptable toxicity occurred in study patient nos. 1–15. The total 1250 mg daily dose was divided into two and administered twice daily (BID) in the subsequent patients (nos. 16–45) to better adjust dosing for the short blood half-life of vatalanib; 500 mg (two tablets) were taken in the morning and 750 mg (three tablets) in the evening ∼12 h apart each day.

### Study procedures

Adverse events were recorded and physical examination was performed at each visit performed at 2- to 8-week intervals. Treatment was interrupted if patients exhibited Common Toxicity Criteria (CTC) grade 4 hypertension, ataxia or dizziness, or neutropenia; grade 3 transaminase or bilirubin elevation, ataxia or dizziness >10 days’ duration (related to vatalanib), or any other grade 3 or 4 toxicity other than nausea or vomiting; grade ⩾2 proteinuria (⩾1.0 g) or haematuria; serum creatinine ⩾2.0 × ULN; or thrombocytopenia with platelet count ⩽25 × 10^9^ l^–1^. If toxicities resolved to CTC⩽grade 1 with ANC ⩾1.5 × 10^9^ l^–1^ and platelets ⩾100 × 10^9^ l^–1^, the dose was scheduled to be reduced to 1000 mg for the first episode of toxicity and to 750 mg for the second. Patients who exhibited a third episode of toxicity or had treatment delayed ⩾3 weeks had vatalanib administration discontinued.

Tumour response was assessed using RECIST 1.0 (Therasse *et al*, 2000). Criteria required for determining complete response (CR) or PR had to be present ⩾4 weeks. Computed tomography or magnetic resonance imaging was performed at baseline, on study week 4, 8, 12 and every 8 weeks thereafter. The same imaging method was required to be used in response assessments. Serum chemistry was performed at baseline and thereafter at 1- to 4-week intervals, and serum coagulation panel at baseline and on week 4. Chest radiographs were obtained at baseline, week 4 and week 12; and electrocardiograms at baseline, week 4, week 12 and every 8 weeks thereafter. Urinalyses were performed at baseline, week 2, week 4 and every 4 to 8 weeks thereafter. *KIT* and *PDGFRA* mutation analyses were not required for study entry.

### Statistical analysis

The first 15 patients who were the subject of the prior report (Joensuu *et al*, 2008) are included in the current analysis that uses a two-stage design (*n*_1_=15, *n*_2_=30). Subsequent analyses were performed separately in the subgroups of patients with and without prior sunitinib therapy. We were interested to detect a clinical benefit rate (the primary end point) different from 0.20. Accepting an *α*=0.05 (type I error), 15 patients in the first stage and 30 patients in the second stage (45 patients overall) would provide 81% power at *P*=0.38 based on a one sample two-stage design (O’Brien and Fleming use function) with unequal sample sizes per stage.

Response (CR+PR) and SD rates were calculated by intent to treat. Response duration and duration of SD were calculated from the date of first occurrence to the date of disease progression, death or last follow-up. Disease was required to fulfil the criteria of SD for a minimum duration of 3.0 months in order to be categorised as SD. Clinical benefit was defined as confirmed or unconfirmed CR or PR, or SD ⩾3.0 months.

Frequency tables were analysed using the *χ*^2^ test. Cumulative survival was estimated using the Kaplan–Meier life-table method, and survival between groups was compared using the log-rank test. Time to progression was defined as the time interval from the date of treatment initiation to the date of PD or death due to cancer, and patients who were alive without disease progression were censored on the date of last follow-up. All *P*-values are two-sided.

## Results

Forty-five patients with advanced GIST were enrolled between August 2004 and November 2007 in the study. The patient population was predominantly (76%) male and had a median age of 61 years (range, 19–82), and most had a good performance status (0 or 1; Table 1). Most had received imatinib at least 800 mg daily with either PR or SD as their best response. Only one (2.2%) patient did not tolerate imatinib. Nineteen (42.2%) had received prior sunitinib in addition to prior imatinib.

Patients underwent treatment with vatalanib for a median of 3.1 months (range, 0.03–44.0 months). Forty-three (95.6%) patients have completed the study, 39 (90.7%) of whom had PD upon discontinuation. The remaining four patients discontinued the study due to sudden death (*n*=1), adverse effect (*n*=2) or withdrawal of consent (*n*=1). Two patients had an ongoing SD and continued to receive vatalanib 21.8 and 35.6 months after study entry.

**Figure 1:**
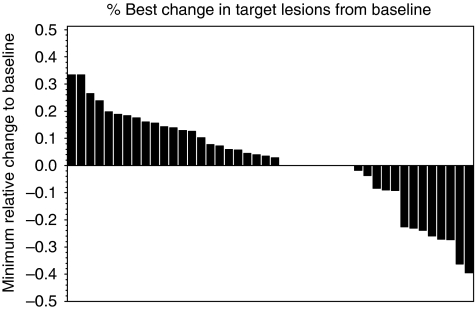
A waterfall plot showing the minimum relative change in tumour mass compared with baseline in 45 GISTs treated with vatalanib.

Two (4.4%) patients achieved a confirmed PR as best response (three further patients had unconfirmed PR), and 16 (35.6%) had SD lasting for ⩾3 months after study entry (Figure 1). Overall, 18 patients had a clinical benefit (40.0% 95% confidence interval (CI), 25.7–54.3%). The PRs lasted for 9.6 and 39.4 months, and the median duration of the 16 SDs was 12.5 months (95% CI, 9.1–25.8 months; range, 6.0–35.6+ months). Twelve (46.2%) out of the 26 patients who did not receive prior sunitinib achieved either a PR (*n*=2, 7.7%) or SD (*n*=10, 38.5%) compared with six (31.6%, all SDs) out of the 19 patients who had received prior imatinib and sunitinib (*P*=0.324). The median duration of SD among the patients who had not received prior sunitinib was 9.9 months (range, 6.3–33.8 months), and among those with prior both imatinib and sunitinib therapy 19.7 months (range, 6.0–36.5+) months.

**Figure 2:**
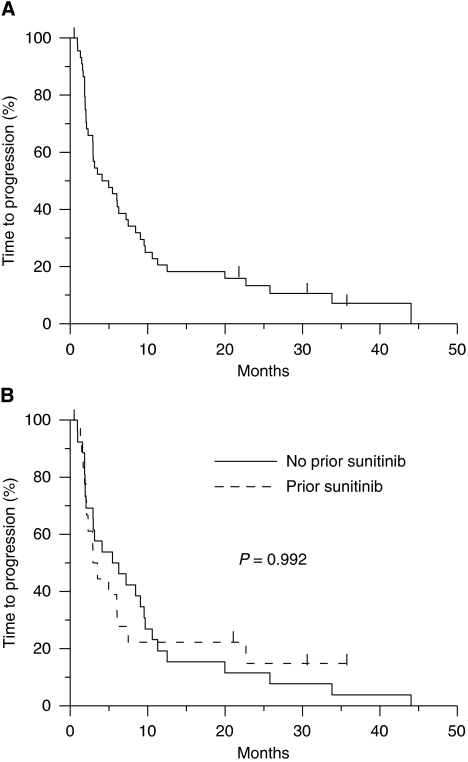
(Upper panel) Time to progression (all patients). (Lower panel) Time to progression of 26 patients treated with prior imatinib only and 19 patients with prior imatinib and sunitinib. Patients censored are indicated with a bar.

The median TTP was 4.5 months (95% CI, 2.9–8.4 months). The median TTP was 5.8 months (95% CI, 2.9–9.5 months) in the subset of patients who had not received prior sunitinib and 3.2 months (95% CI, 2.1–6.0 months) among those exposed to prior imatinib and sunitinib (*P*=0.992; Figure 2).

Vatalanib 1250 mg per day was generally well tolerated, and no permanent dose reductions were required. The dose was temporarily reduced in 39 (87%) out of the 45 patients for 1 to 64 days as per protocol due to an adverse event or laboratory test abnormality (*n*=4). After the reductions, the dose was returned back to 1250 mg per day.

Adverse events reported for ⩾2 patients, regardless of the CTC grade, are presented in Table 2. The most frequently reported adverse events were hypertension (*n*=13, 28.9%), nausea (*n*=13, 28.9%), dizziness (*n*=11, 24.4%), proteinuria (*n*=9, 20.0%), abdominal pain (*n*=8, 17.8%) and diarrhea (*n*=8, 17.8%). Most adverse events were mild (grade 1 or 2); only 23 severe (grade 3 or 4) adverse events were recorded during the study. Of these, four were grade 4 in severity (hypercalcemia, pain, pulmonary embolism, sudden death; *n*=1 for each), of which only pulmonary embolism was possibly related to study medication. Only one patient was diagnosed with hypothyroidism and none with hand–foot skin reaction.

## Discussion

Vatalanib turned out to be active as second-line or third-line treatment of advanced imatinib-resistant or imatinib and sunitinib-resistant GIST. To our knowledge, the current study is the only one that has addressed vatalanib in the treatment of GIST. The results of the present expanded cohort are consistent with those of the first 15 patients included in the series, in which subset only prior imatinib was allowed before study entry (Joensuu *et al*, 2008). Although the PR rate was low (4.4%), many patients (35.6%) had SD lasting for ⩾6 months, suggesting substantial vatalanib activity. Of note, 6 (31.6%) out of the 19 patients who progressed both on imatinib and sunitinib achieved SD that lasted for a median of 19.7 months (range, 6.0–36.5 months), suggesting that vatalanib has clinically meaningful activity in some patients whose disease no longer responds to imatinib and sunitinib. In the subset of patients who had been exposed only to prior imatinib (*n*=26), the response rate to vatalanib was 7.7% and further 38.5% of patients had SD. These figures resemble remarkably closely those obtained with sunitinib in a comparative patient population. PR rates ranging from 7 to 13% and SD rates raging from 33 to 59% were achieved with sunitinib in patients who had imatinib-resistant GIST or who were intolerant to imatinib (Maki *et al*, 2005; Demetri *et al*, 2006; George *et al*, 2009). Similarly, the median TTP achieved in the current study with vatalanib in this patient group (5.8 months) is roughly similar to the 6.3 months achieved with sunitinib (Demetri *et al*, 2006). Yet, comparisons between different series need to be interpreted with caution.

Vatalanib was in general very well tolerated. The most frequent adverse effects were rarely graded severe. Hypertension occurred in 28.9% of the patients, but it was generally easily manageable. No patient with severe dizziness, nausea or proteinuria was reported, and none of these adverse effects resulted in treatment discontinuation or dose reduction. Dizziness typically occurred for a few hours after taking vatalanib, and could be managed either by dividing the daily dose or by administration of vatalanib towards the evening. Only four grade 4 adverse events were recorded during the study, of which one, pulmonary embolism, was possibly vatalanib related. One patient died unexpectedly during the study, but the cause of death was probably unrelated to the study drug. We recorded no patients with hand–foot skin reaction or mucositis, which are common adverse effects of sunitinib, and hypothyroidism and fatigue were also rare and classified mild in severity. Safety of vatalanib thus appears to compare well with that of sunitinib or the higher doses of imatinib (Verweij *et al*, 2004), although no direct comparative data are available.

Vatalanib may not be developed further in clinical trials, mainly because it did not improve survival in combination with chemotherapy in two large prospective randomised trials (CONFIRM-1 and CONFIRM-2) carried out in advanced colorectal cancer (Hecht *et al*, 2011; Van Cutsem *et al*, 2011). This may be unfortunate considering the few treatment options available for imatinib-resistant GIST, since not all GIST patients tolerate sunitinib well, and effective and well-tolerated third-line treatments are urgently needed. Furthermore, the present results are of particular interest and important from a hypothesis-generating point of view as well. Vatalanib is a narrow-spectrum TKI (Karaman *et al*, 2008), and yet, it had activity in imatinib and sunitinib-resistant GIST despite that both imatinib and sunitinib interact with the kinome more broadly than vatalanib. This suggests that KIT (and PDGFRA) still remains a viable target in imatinib and sunitinib-resistant GIST. Kinase inhibition will likely result in the greater toxicity the more kinases are inhibited. Since inhibition of many kinases may thus not always be required for significant clinical activity in imatinib-resistant GIST, it may be possible to develop well tolerated and yet effective inhibitors for imatinib-resistant GIST. The novel switch pocket inhibitors may be one such approach (Heinrich *et al*, 2010).

We did not collect tissue for *KIT* and *PDGFRA* mutation analyses, which may be considered a limitation of the study. This limitation may, however, be minor, because acquired *KIT* mutations are common in imatinib-resistant GIST reducing the applicability of the primary tumour mutation analysis (Heinrich *et al*, 2003), and because recent studies show that multiple acquired mutations are often present in imatinib-resistant GIST (Wardelmann *et al*, 2006; Liegl *et al*, 2008). In imatinib-resistant disease, the number of acquired *KIT* mutations detected may be dependent on the number of biopsies taken, several acquired mutations are often detected in the same individual in different metastases, and sometimes even one metastasis may contain more than one resistance mutation (Liegl *et al*, 2008). Therefore, unlike in the first-line treatment of advanced GIST or in the adjuvant setting where mutation analysis is of importance, mutation analysis appears to have limited value in selection of systemic treatment for imatinib-resistant GIST.

Vatalanib was administered QD in the first part of the study (patients 1–15), but BID in the expansion part, because vatalanib has a short half-life (4.5 h) and the twice daily administration might have increased efficacy. We did not, however, observe a significant difference in efficacy between the two dosing regimens, but this may have been confounded by the amended patient selection criteria, where prior sunitinib was allowed in the expansion part of the study. In a phase I study where 150–1000 mg of vatalanib was given BID to patients with solid tumours (Thomas *et al*, 2005), its exposure increased with dosing up to 500 mg BID and reached a plateau at higher doses, suggesting that total daily doses ⩾1000 mg are optimal for clinical trials.

We conclude that vatalanib is active in patients who have imatinib-resistant GIST or imatinib and sunitinib-resistant GIST. Although only a few PRs were achieved, several of the SDs were durable. The overall efficacy results resemble those obtained with sunitinib, the only currently approved second-line therapy for imatinib-resistant GIST. Of note, vatalanib was generally well tolerated, which is in line with its narrow kinase interaction spectrum. The results suggest that relatively narrow-spectrum, well-tolerated TKIs may be effective in the treatment of imatinib and sunitinib-resistant GIST, and that mutated KIT may thus frequently remain a key target in advanced GIST that has become resistant to one or more TKIs.

## Figures and Tables

**Table 1:** Patient characteristics

	**Number of patients (*N*=45)**
**Characteristic**	***n* (%)**
Age, median (range)	61 (19–82)
	
*Gender*	
Male	34 (75.6)
Female	11 (24.4)
	
*WHO performance status at baseline*	
0	24 (57.1)
1	17 (40.5)
2	1 (2.4)
N.A.	3
	
*Primary site of lesion*	
Small intestine	18 (40.0)
Stomach	18 (40.0)
Rectum	2 (15.6)
Other	7 (4.4)
	
*Metastatic sites (*n=*102)*	
Liver	39 (38.2)
Peritoneum/omentum	19 (18.6)
Intra-abdominal, other	23 (22.5)
Other/unknown	18 (17.6)
Ascites/pleural fluid	3 (2.9)
	
*Highest prior imatinib dose, mg per day*	
400	2 (4.4)
600	6 (13.3)
800	36 (80.0)
1000	1 (2.2)
	
*Best prior response to imatinib*	
CR	0 (0.0)
PR	17 (43.6)
SD	15 (38.5)
PD	7 (17.9)
N.A.	6
	
*Prior treatment with sunitinib*	
Yes	19 (42.2)
No	26 (57.8)
	
*Best prior response to sunitinib (*n=*19)*	
CR	0 (0.0)
PR	3 (16.7)
SD	10 (55.6)
PD	5 (27.8)
N.A.	1

Abbreviations: WHO=World Health Organization; N.A.=data not available; CR=complete response; PR=partial response; SD=stable disease; PD=progressive disease.

**Table 2:** Adverse events occurring in at least 4% of patients

	**Toxicity by grade**			
	**Grade 1 or 2 (*N*=45)**	**Grade 3 (*N*=45)**	**Grade 4 (*N*=45)**	**Total (*N*=45)**
**Adverse event**	***n* (%)**	***n* (%)**	***n* (%)**	***n* (%)**
Hypertension^a^	8 (17.8)	4 (8.9)	0	13 (28.9)
Nausea	13 (28.9)	0	0	13 (28.9)
Dizziness	11 (24.4)	0	0	11 (24.4)
Proteinuria	9 (20.0)	0	0	9 (20.0)
Abdominal pain	6 (13.3)	2 (4.4)	0	8 (17.8)
Diarrhea	7 (15.6)	1 (2.2)	0	8 (17.8)
Asthenia	6 (13.3)	1 (2.2)	0	7 (15.6)
Dyspepsia	5 (11.1)	0	0	5 (11.1)
Pyrexia	5 (11.1)	0	0	5 (11.1)
Upper abdominal pain	5 (11.1)	0	0	5 (11.1)
Vomiting	5 (11.1)	0	0	5 (11.1)
Fatigue	4 (8.9)	0	0	4 (8.9)
Headache	3 (6.7)	1 (2.2)	0	4 (8.9)
Pain	3 (6.7)	0	1 (2.2)	4 (8.9)
Anaemia	3 (6.7)	0	0	3 (6.7)
Constipation	3 (6.7)	0	0	3 (6.7)
Hyperhidrosis	3 (6.7)	0	0	3 (6.7)
Visual disturbance	3 (6.7)	0	0	3 (6.7)
ALT increased	0	2 (4.4)	0	2 (4.4)
Anorexia	2 (4.4)	0	0	2 (4.4)
Arthralgia	2 (4.4)	0	0	2 (4.4)
AST increased	1 (2.2)	1 (2.2)	0	2 (4.4)
Dysphonia	2 (4.4)	0	0	2 (4.4)
Epitaxis	2 (4.4)	0	0	2 (4.4)
Insomnia	2 (4.4)	0	0	2 (4.4)
Myalgia	2 (4.4)	0	0	2 (4.4)
Oedema, peripheral	2 (4.4)	0	0	2 (4.4)
Pulmonary embolism	0	1 (2.2)	1 (2.2)	2 (4.4)
Polyuria	2 (4.4)	0	0	2 (4.4)
Weight decreased	2 (4.4)	0	0	2 (4.4)

Abbreviations: ALT=alanine aminotransferase; AST=aspartate aminotransferase; CTC=Common Toxicity Criteria.

a CTC grade missing for one event.
